# Breast cancer genomes from *CHEK2* c.1100delC mutation carriers lack somatic *TP53* mutations and display a unique structural variant size distribution profile

**DOI:** 10.1186/s13058-023-01653-0

**Published:** 2023-05-09

**Authors:** Marcel Smid, Marjanka K. Schmidt, Wendy J. C. Prager-van der Smissen, Kirsten Ruigrok-Ritstier, Maartje A. C. Schreurs, Sten Cornelissen, Aida Marsal Garcia, Annegien Broeks, A. Mieke Timmermans, Anita M. A. C. Trapman-Jansen, J. Margriet Collée, Muriel A. Adank, Maartje J. Hooning, John W. M. Martens, Antoinette Hollestelle

**Affiliations:** 1 Department of Medical Oncology, Erasmus MC Cancer Institute, Rotterdam, The Netherlands; 2 Division of Molecular Pathology, The Netherlands Cancer Institute - Antoni van Leeuwenhoek Hospital, Amsterdam, The Netherlands; 3 Department of Clinical Genetics, Leiden University Medical Center, Leiden, The Netherlands; 4 Core Facility Molecular Pathology & Biobanking, The Netherlands Cancer Institute - Antoni van Leeuwenhoek Hospital, Amsterdam, The Netherlands; 5 Department of Clinical Genetics, Erasmus University Medical Center, Rotterdam, The Netherlands; 6 Family Cancer Clinic, The Netherlands Cancer Institute - Antoni van Leeuwenhoek Hospital, Amsterdam, The Netherlands

**Keywords:** CHEK2, Whole-genome sequencing, Somatic cancer genome, Mutational landscape, *TP53* mutation, Structural variation, Size distribution, Chromothripsis, Whole-genome duplication, Breast cancer

## Abstract

**Background:**

*CHEK2* c.1100delC was the first moderate-risk breast cancer (BC) susceptibility allele discovered. Despite several genomic, transcriptomic and functional studies, however, it is still unclear how exactly *CHEK2* c.1100delC promotes tumorigenesis. Since the mutational landscape of a tumor reflects the processes that have operated on its development, the aim of this study was to uncover the somatic genomic landscape of CHEK2-associated BC.

**Methods:**

We sequenced primary BC (pBC) and normal genomes of 20 *CHEK2* c.1100delC mutation carriers as well as their pBC transcriptomes. Including pre-existing cohorts, we exhaustively compared CHEK2 pBC genomes to those from *BRCA1*/*2* mutation carriers, those that displayed homologous recombination deficiency (HRD) and ER− and ER+ pBCs, totaling to 574 pBC genomes. Findings were validated in 517 metastatic BC genomes subdivided into the same subgroups. Transcriptome data from 168 ER+ pBCs were used to derive a *TP53*-mutant gene expression signature and perform cluster analysis with CHEK2 BC transcriptomes. Finally, clinical outcome of *CHEK2* c.1100delC carriers was compared with BC patients displaying somatic *TP53* mutations in two well-described retrospective cohorts totaling to 942 independent pBC cases.

**Results:**

BC genomes from *CHEK2* mutation carriers were most similar to ER+ BC genomes and least similar to those of *BRCA1/2* mutation carriers in terms of tumor mutational burden as well as mutational signatures. Moreover, CHEK2 BC genomes did not show any evidence of HRD. Somatic *TP53* mutation frequency and the size distribution of structural variants (SVs), however, were different compared to ER+ BC. Interestingly, BC genomes with bi-allelic *CHEK2* inactivation lacked somatic *TP53* mutations and transcriptomic analysis indicated a shared biology with *TP53* mutant BC. Moreover, CHEK2 BC genomes had an increased frequency of > 1 Mb deletions, inversions and tandem duplications with peaks at specific sizes. The high chromothripsis frequency among CHEK2 BC genomes appeared, however, not associated with this unique SV size distribution profile.

**Conclusions:**

CHEK2 BC genomes are most similar to ER+ BC genomes, but display unique features that may further unravel CHEK2-driven tumorigenesis. Increased insight into this mechanism could explain the shorter survival of *CHEK2* mutation carriers that is likely driven by intrinsic tumor aggressiveness rather than endocrine resistance.

**Supplementary Information:**

The online version contains supplementary material available at 10.1186/s13058-023-01653-0.

## Background

The *CHEK2* c.1100delC mutation, leading to premature translation termination, was discovered to be the first moderate-risk breast cancer (BC) susceptibility allele in 2002 [1, 2]. Women who carry this germline mutation have a 2.3-fold increased risk to develop BC during their life time compared with the general population [3, 4]. BCs from *CHEK2* mutation carriers are mostly of the luminal/ER+ subtype and are diagnosed at a younger age than sporadic BCs (median age of 50 vs 60 years) [5–8]. Furthermore, BC patients carrying the c.1100delC mutation have increased risk of developing a contralateral BC and a worse survival compared to sporadic BC patients although resistance to either endocrine or chemotherapy does not appear to play a role herein [5, 8–12]. To provide tailored prevention and treatment strategies for *CHEK2* mutation carriers, it is important to unravel the biological mechanism that *CHEK2* c.1100delC exploits to drive tumorigenesis.

Similar to BRCA1 and BRCA2, CHEK2 operates in the DNA damage response (DDR) pathway. Once activated, CHEK2 is able to phosphorylate more than 20 different effector proteins involved in DNA repair, cell cycle regulation, TP53 signaling and apoptosis (e.g., BRCA1, CDC25A, TP53, and PML) [13]. Considering the central role of CHEK2 in these pathways and the merely moderate BC risk the c.1100delC mutation confers, many of its functions must be redundant in mammary epithelial cells in which CHEK2-associated BCs arise.

Functional studies and mouse models have produced conflicting results [14–18]. One important reason for this is likely the use of either non-human model systems or non-mammary epithelial cell types. Considering the latter, hormonal factors seem to play an important role in the development of BC in women and mice carrying the c.1100delC mutation, since the vast majority of BCSs in women is of the luminal/ER+ subtype [6, 7] and *Chk2* c.1100delC knock-in mice developed tumors preferentially in females [18].

In addition, results from gene expression and copy number (CN) profiling studies on CHEK2-associated BCs have also not provided significant clues regarding CHEK2-driven tumorigenesis [7, 19, 20]. In this respect, next-generation sequencing technology has provided much insight into the mutational processes that operate during tumorigenesis in recent years. For example, mutational profiling has identified the APOBEC-catalyzed cytidine deamination to be a major source of mutation in cancer [21]. Moreover, homologous recombination repair deficiency (HRD), caused by loss of BRCA1 or BRCA2 function, leaves a specific genomic imprint on the DNA characterized by two single base substitution (SBS) signatures (SBS3 and SBS8), one small insertion/deletion (ID) signature (ID6) and two specific structural variant (SV) signatures (SV3 for BRCA1-type cancers and SV5 for BRCA2-type cancers) [22–24]. Interestingly, BCs from women carrying truncating variants in *CHEK2* did not display a dominant HRD-related mutational signature, in contrast to BCs from *BRCA1*, *BRCA2* and *PALB2* mutation carriers, but similar to BCs from *ATM* mutation carriers [25–27]. Both studies on CHEK2- and ATM-associated BCs used exome and targeted sequencing data, limiting resolution and precluding the analyses of larger SVs.

In the current study, we have sequenced the primary tumor and normal genomes of 20 *CHEK2* c.1100delC mutation carriers as well as their tumor transcriptomes. Including pre-existing genomic data, we exhaustively compared CHEK2 primary BC (pBC) genomes to pBC genomes from *BRCA1*/*2* mutation carriers, pBCs that displayed HRD and ER− and ER+ pBCs, totaling to 574 pBC genomes. Findings were validated in 517 metastatic BC (mBC) genomes subdivided into the same subgroups.

## Methods

### Pre-existing genomic data

As part of the International Cancer Genome Consortium’s (ICGC) effort to coordinate large-scale cancer genome studies in tumors from 50 cancer types and/or subtypes, whole-genome sequencing (WGS) data from 560 pBCs were generated [23] which is available from the European Genome-phenome Archive (accession code EGAS00001001178). Information on sample selection and clinical data from this cohort is available in the supplementary data of the original study at https://www.nature.com/articles/nature17676.

The Center for Personalized Cancer Treatment (CPCT), involving more than 40 Dutch hospitals, aims to provide personalized cancer treatment through WGS of patient’s mBC biopsies at Hartwig Medical Foundation (HMF). The resulting WGS and clinical data included 517 mBCs suitable for our analyses at the time of our data request (September 2019, DR-085). Inclusion criteria for CPCT were described previously [28].

### Whole-genome sequencing

*CHEK2* c.1100delC mutation carriers from which we had fresh-frozen pBC tissue as well as corresponding normal tissue or blood were identified retrospectively from the tissue banks of the Erasmus MC Cancer Institute and Netherlands Cancer Institute and their family clinics. For inclusion, BCs required a tumor percentage ≥ 40% and genomic DNA of sufficient quantity (≥ 500 ng) and quality (A260/A280 = 1.8–2.0 and DNA length ≥ 10 kb) for WGS, as did the corresponding normal material. Upfront, presence of the *CHEK2* c.1100delC mutation in tumor-normal pairs was verified with a custom-made Taqman genotyping assay (Thermo Fisher, Waltham, MA) as described elsewhere [3]. It was also verified that tumor-normal pairs were from the same individual by short tandem repeat analysis using the PowerPlex16 System (Promega, Madison, WI) before sending genomic DNA from the remaining 20 *CHEK2* c.1100delC mutation carriers to HMF (Amsterdam, the Netherlands) for WGS, subsequent genome alignment and variant calling as described previously for the CPCT cohort [28]. The resulting genomic data revealed one homozygous carrier and 19 heterozygous carriers of which eleven displayed loss and six retained the wild-type *CHEK2* allele. Two had lost the mutant allele and were excluded from all further analyses, totaling to 18 CHEK2 pBC genomes (patient and tumor characteristics in Additional file 1: Table S1).

In addition to the above, we also selected genomic DNA from three pBC-normal pairs from the Erasmus MC Cancer Institute that were previously included in the 560 pBCs from the ICGC (*i.e.,* PD4604, PD4607 and PD13620) [23]. After WGS at HMF, these samples were also processed using the same pipeline as the CHEK2 pBCs and CPCT mBCs to compare WGS data from HMF versus ICGC pipelines.

### Subgroups for analysis

**Fig. 1:**
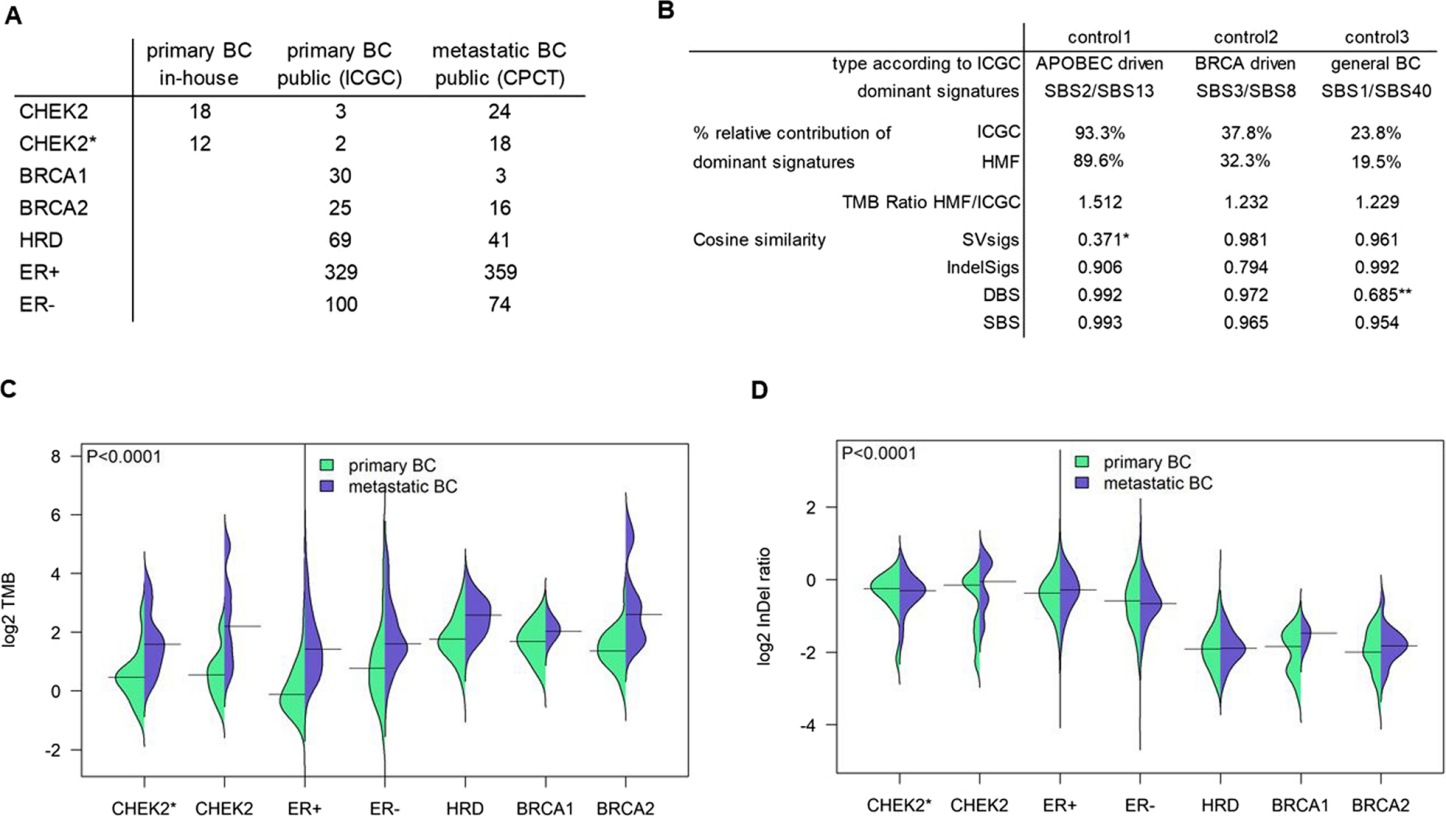
Cohorts, controls, TMB and ID ratio. **A** Numbers of samples by cohort. Four ICGC samples with *CHEK2* mutations other than c.1100delC were excluded from the dataset and subsequent analyses. **B** Results of samples analyzed on both HMF and ICGC pipelines. *only 17 SVs and **only 18 DBS for signature calling. Distributions of C TMB and D ID ratio by group. Horizontal line shows median TMB. *P*-values are from Kruskal–Wallis comparison of the primary BC subgroups

In addition to the 18 CHEK2 pBC genomes we generated for the current study, the ICGC pBC cohort also contained three *CHEK2* c.1100delC pBC genomes of which two displayed loss of the wild-type allele, totaling to 21 CHEK2 pBC genomes. Four ICGC pBCs with *CHEK2* mutations other than c.1100delC were excluded from all further analyses. Analyses were performed separately for the CHEK2 group, which contained all CHEK2 pBCs (*n* = 21) and the CHEK2* group, which only contained CHEK2 pBCs with bi-allelic *CHEK2* inactivation (*n* = 14; Fig. 1A).

To compare CHEK2 pBC genomes to the remaining 553 pBC genomes from ICGC [23], we considered five additional groups: 1) germline *BRCA1* or 2) *BRCA2* mutation carriers that display loss of the wild-type allele, 3) samples not in groups 1 or 2 that have an HRD phenotype, and 4) ER− and 5) ER+ samples not in groups 1–3 (numbers per subgroup detailed in Fig. 1A). Some analyses were performed with an HRD+ group in which samples of groups 1–3 were combined.

Findings from analyses on pBC subgroups were validated in the 517 mBC genomes from the CPCT cohort which was subdivided into the same seven groups mentioned above (numbers per subgroup detailed in Fig. 1A).

### Bioinformatics analyses

Tumor mutational burden (TMB) was defined as the number of somatic variants (*i.e.*, SNVs, MNVs and IDs) per million mappable bases (set at 2,858,674,661/10^6^) [29]. R v4.0.3 was used in conjunction with several packages for a range of analyses on the BC genomes: MutationalPatterns v3.0.1 for assigning mutational signatures [30], CHORD for classifying BRCA1-type HRD, BRCA2-type HRD and homologous recombination repair proficient (HRP) tumors [31], dndscv v0.1.0 for identifying driver genes [32], Facets v0.6.1 for detecting whole-genome duplication (WGD) [33], Shatterseek v0.4 for detecting chromothripsis [34] and kmlShape v0.9.5 for calculating the Fréchet distance (https://CRAN.R-project.org/package=kmlShape).

HRDetect calls (*i.e.,* HRD or HRP) for the ICGC pBCs and CPCT mBCs were publicly available [31]. For WGD, the fraction of segments that showed a major CN ≥ 2 was calculated per sample. Since a histogram of all sample fractions showed a clear bimodal distribution, the cut-point for calling a sample WGD was established at the lowest point between the two peaks. If Shatterseek identified at least one chromothripsis region the sample was labeled positive. SV signatures were called as previously described [29]. To compare density profiles using the Fréchet distance, we first established the baseline density profile of all samples per SV type (*i.e.,* inversions, deletions and tandem duplications (TDs)). We then used the density profile of a subgroup (*e.g.,* inversions of the CHEK2* group), sampled these data 100 times with replacement (bootstrapping) and calculated the Fréchet distance of each bootstrap to the baseline profile of all samples. Lastly, the distribution of 100 distances of a subgroup was compared to the distribution of distances of another subgroup using a t-test.

### RNA sequencing

Total RNA was isolated from the same frozen tumor tissue for the 20 *CHEK2* c.1100delC mutation carriers using RNA-Bee. After clean up and DNase I treatment, 1 µg of RNA was send to Novogene (Cambridge, UK) for Illumina RNA sequencing using a ribosomal RNA depletion method. Raw sequence files were mapped to GRCh38 using STAR v2.6.1d [35]. Sambamba v0.7.0 [36] was used to mark duplicates and index the resulting BAM files. Raw read counts for genes were obtained with featureCounts v1.6.3 [37] and normalized using GeTMM [38]. RNA sequencing data from the ICGC cohort was processed similarly [39], merged with the RNA sequencing data of the CHEK2 cohort and adjusted for batch effects using ComBat [40]. Linear regression models were used to extract differentially expressed genes between groups. Hierarchical clustering of samples was achieved by first constructing a correlation-matrix of sample vs. sample based on these differentially expressed genes.

### Clinical cohort

The two clinical cohorts totaled to 942 independent pBC cases and could be subdivided into our previously well-described retrospective cohorts of 760 lymph-node negative treatment-naïve ER+ BC patients (prognostic cohort) and 323 hormone-naïve ER+ BC patients treated with first-line tamoxifen for recurrent disease (predictive cohort) [41]. The complete *TP53* coding sequence from these patient’s pBCs was evaluated for genetic alterations by Sanger sequencing (primers available upon request). *CHEK2* c.1100delC status was again determined using a previously published custom-made Taqman genotyping assay (Thermo Fisher) [3]. Loss of the wild-type *CHEK2* c.1100delC allele was evaluated by deep sequencing of a 144-bp nested-PCR amplicon encompassing the *CHEK2* mutation (primers derived from Taqman genotyping assay) on an Ion Torrent PGM (Thermo Fisher) and taking tumor cell percentage into account.

### Statistics

Categorical data were evaluated using Pearson’s χ^2^ test or Fisher’s exact test (when too few expected events). For continuous variables, a Mann–Whitney or Kruskal–Wallis test was performed. For time-to-event data, the logrank test and Cox proportional hazards models were used to compare disease-free survival between groups. Overall response (*i.e.,* complete response, partial response and stable disease > 6 months vs. stable disease < 6 months and progressive disease) to first-line tamoxifen treatment for recurrent disease between groups was evaluated using logistic regression analysis. Multivariable analyses included all clinicopathological variables that displayed significant associations in univariable analyses. Other tests are indicated where applicable. All statistical tests were two-sided and considered statistically significant when *P* < 0.05. Stata 13.0 (StataCorp, College Station, TX) and R v4.0.3 were used to perform analysis. The Hochberg procedure was used to correct *P*-values for multiple hypothesis testing when appropriate.

## Results

### Comparison of sequencing pipelines

The CHEK2 pBC cohort and CPCT mBC cohort were sequenced and processed by HMF, while the ICGC pBC cohort was sequenced and processed differently [23]. Existing systematic differences between the two pipelines could confound cross-cohort comparisons. Therefore, we resequenced three tumor-normal pairs from the ICGC dataset at HMF. Comparison of these pairs showed (Fig. 1B) that the HMF pipeline called more variants, reflecting the higher tumor sequence coverage by HMF (90X) versus ICGC (40X). However, the global nature and patterns of the variants, condensed in the various mutational signatures, were very comparable between the pipelines. In fact, cosine similarities between the three pairs of SBS, double base substitution (DBS), ID and SV signatures were > 0.90 for 9/12 comparisons), while 2/3 comparisons with a cosine similarity < 0.90 could be explained by a low number of DSBs and SVs. If an underlying systematic bias existed between the two pipelines, overall low cosine similarities would be observed. Therefore, we were confident to perform comparative analyses between the cohorts and further subgroup pBC and mBC genomes into the following seven groups: CHEK2, CHEK2* (*i.e.,* only CHEK2 BCs with bi-allelic CHEK2 inactivation), BRCA1, BRCA2, HRD, ER− and ER+ . Subgrouping is further detailed in the Methods (numbers per subgroup listed in Fig. 1A). Additional file 1: Table S2 contains an overview of genomic events in all samples.

### TMB and ID ratio

Notwithstanding the higher rate of variants called by the HMF pipeline, pBC genomes from *CHEK2* mutation carriers had a lower TMB than HRD+ pBC genomes (Fig. 1C). Distributions over all groups were significantly different (*P* < 1.0*10^−4^), with false discovery rate adjusted post hoc comparisons showing significantly lower median TMB for CHEK2* (1.37) compared to BRCA1 (3.20, *P*_adj_ = 6.5*10^−3^), BRCA2 (2.55, *P*_adj_ = 0.049) and HRD (3.40, *P*_adj_ = 1.3*10^−3^), but not compared to ER− or ER+ pBCs (1.71 and 0.92, *P*_adj_ > 0.05). Consistent with tumorigenic progression and treatment-induced selection [29], median TMB was 2.3-fold higher in the mBC compared with the pBC cohort. However, similar differences in TMB were observed among mBC groups (Fig. 1C, P < 1.0*10^−4^) with only CHEK2* mBCs showing a significant lower TMB compared to HRD mBCs (*P*_adj_ = 0.013) in the post hoc comparison.

The ratio of insertions over deletions (Fig. 1D) showed a similar distribution in CHEK2 pBCs compared with ER− and ER+ pBCs, while being significantly higher compared to BRCA1, BRCA2 and HRD pBCs (*P* < 1.0*10^−4^ over all groups; FDR-adjusted post hoc comparisons *P*_adj_ = 1 for ER+ , *P*_adj_ = 0.36 for ER− , and *P*_adj_ < 0.0001 for HRD+ vs CHEK2* pBC). Again, these findings were validated in the mBC cohort (*P* < 1.0*10^−4^ over all groups; post hoc comparisons were significant for CHEK2* vs. BRCA2 and HRD, both *P*_adj_ < 1.0*10^−4^). Lastly, in contrast to the TMB, the ID ratio was not significantly increased from the primary to metastatic setting, except within ER+ BC (median of 0.77 vs. 0.82, *P* = 0.02) though the effect size is very modest.

Thus, in terms of TMB and ID ratio, BC genomes of *CHEK2* c.1100delC carriers are most similar to ER− and ER+ and least similar to HRD+ BC genomes.

### Mutational signatures

**Fig. 2:**
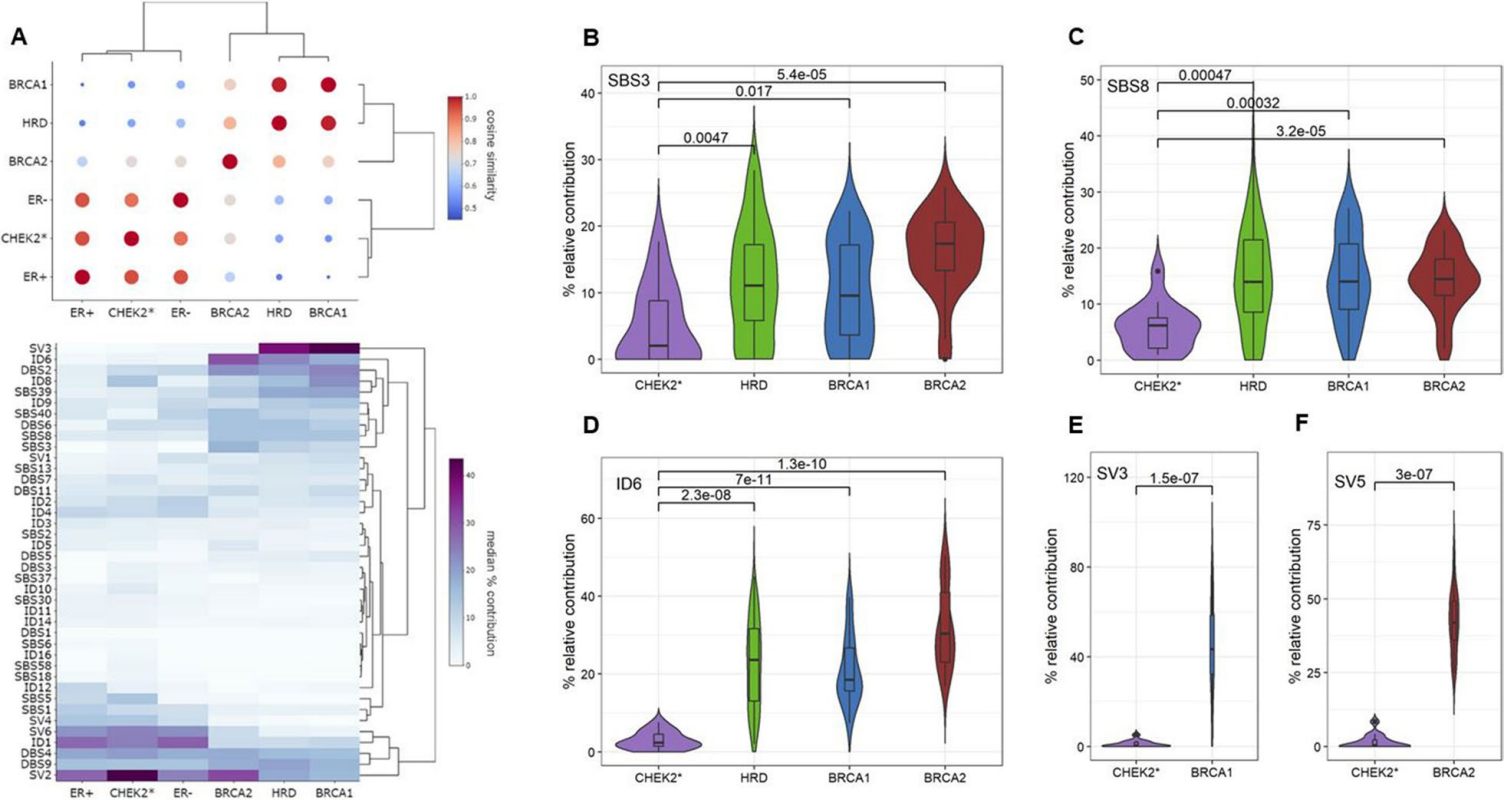
Mutational signatures in primary BC subgroups. **A** Cosine similarity coefficients (top) and hierarchical clustering (bottom) of BC subgroups based on the median % relative contribution of 40 SBS, DSB, ID and SV signatures. Percentage relative contribution of mutational signatures associated with the HRD phenotype: **B** SBS3, **C** SBS8, **D** ID6, and signatures associated with **E** BRCA1 (SV3) and **F** BRCA2 (SV5). *P*-values are based on Mann–Whitney test

To reveal the mutational processes operating during breast tumorigenesis in *CHEK2* c.1100delC mutation carriers, we determined the percentage relative contribution (%rc) of each of the known 67 SBS, 11 DBS, 18 ID and 6 SV signatures [23, 24]. Out of these 102 signatures, 13 SBS, 9 DSB, 13 ID and 5 SV signatures had ≥ 5% rc in ≥ 2 CHEK2 BC genomes (Additional file 1: Tables S3-6). For these 40 more profound signatures, we calculated the median %rc of each subgroup and constructed a condensed overview showing CHEK2* pBCs were least similar to HRD+ and most similar to ER+ pBCs (Fig. 2A). This observation was replicated in the mBC cohort, showing CHEK2* mBCs clustering closest to ER+ mBCs using 39/102 signatures with ≥ 5% rc in ≥ 2 CHEK2 mBCs (Additional file 2: Figure S1).

Since CHEK2 BCs as a group were least similar to HRD+ BCs, but CHEK2 is known as a central player in the DDR, we next evaluated whether each individual CHEK2 BC displayed HRD using classifiers CHORD and HRDetect [31, 42]. Both models use specific features in WGS data (*e.g.,* mutational signatures, but also additional characteristics) to distinguish HRD from HRP genomes. Results showed only one of the 21 CHEK2 pBCs displaying HRD, but this pBC had retained the wild-type *CHEK2* allele. Again, only one of the 24 CHEK2 mBCs displayed HRD, but this CHEK2* mBC patient carried an additional *BRCA2* mutation. Finally, we also evaluated individual mutational signatures associated with HRD: SBS3, SBS8, ID6, SV3 and SV5 [22–24] among CHEK2 BCs as a group. However, CHEK2* pBCs showed a significant lower median %rc of these signatures (Fig. 2B–F) compared with HRD+ pBCs, clearly indicating that CHEK2 BCs do not display the obvious mutational scars typical of HRD.

Because *CHEK2* c.1100delC is a moderate-risk allele with lower penetrance than *BRCA1* and *BRCA2* mutations, CHEK2 pBCs might have an intermediate HRD phenotype. However, comparison of SBS3, SBS8, ID6, SV3 and SV5 in CHEK2* versus ER+ BCs did not show a significant difference in the median %rc for these signatures (Additional file 1: Tables S3-6; Additional file 2: Figure S2A). This is consistent with overall mutational signatures of CHEK2 BC genomes being most similar to ER+ BCs. Although we did find significant increases in SBS37, SBS58, ID8, ID10 and ID16 in CHEK2* vs. ER+ pBCs (Additional file 1: Tables S3-6; Additional file 2: Figure S2B), this was not replicated among mBCs. In fact, we identified no significant differences for any of the 102 SBS, DBS, ID or SV signatures between CHEK2 and ER+ mBC genomes. Thus, CHEK2 BCs do not show any evidence for HRD and, based on mutational signatures, are indistinguishable from ER+ BC genomes.

### Somatic BC drivers

**Fig. 3:**
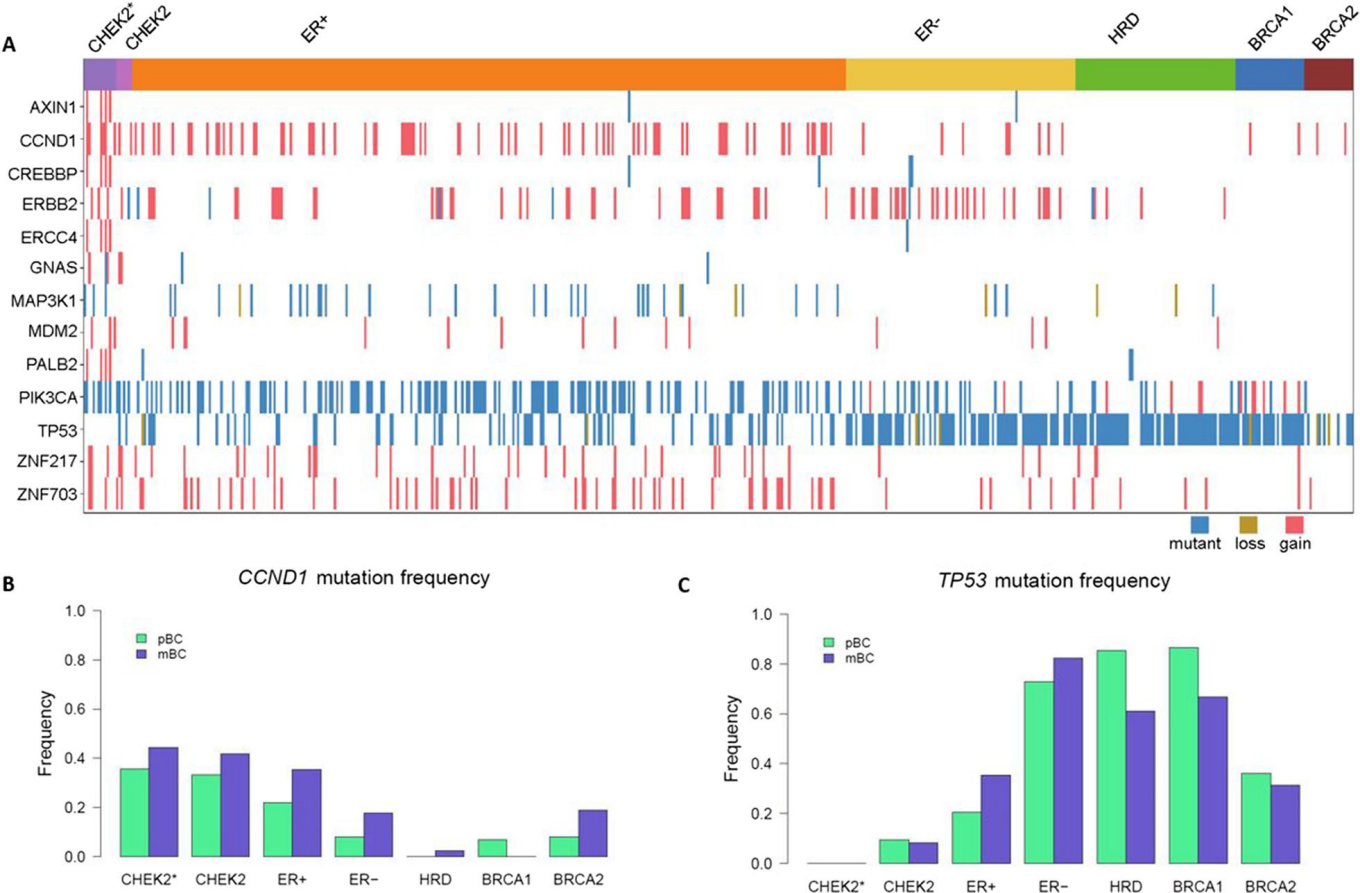
Mutation frequencies of 13 known BC driver genes among subgroups. **A** Oncoplot for *TP53* and 12 known BC driver genes with a mutation frequency > 20% in CHEK2 pBC genomes by subgroup. wt indicates wild-type; mut, any amino-acid changing variant; del, copy number loss; amp, copy number gain. **B** Frequency of *CCND1* and **C** *TP53* mutations by subgroup

We applied the dN/dS method to CHEK2 pBCs, but identified no CHEK2-specific BC driver genes. Therefore, we evaluated the mutation frequency of 94 known somatic BC driver genes [23]. In CHEK2 pBCs, 42 of these 94 driver genes were found mutated (combining protein-changing variants and CN alterations; Additional file 1: Table S7). Interestingly, none of the 14 CHEK2* pBCs harbored a *TP53* mutation (*TP53* and genes > 20% mutated in CHEK2* pBC shown in Fig. 3A), while we expected a mutation frequency similar to ER+ pBCs. Next, we compared the driver mutation frequency between CHEK2 pBCs and the other subgroups and repeated this in mBCs. Combining the results, only *CCND1* (lower frequency in HRD+ ; *P*_adj_ = 3.6*10^−3^ for pBC and *P*_adj_ = 0.010 for mBC) and *TP53* (higher frequency in HRD+ and ER− ; *P*_adj_ = 8.0*10^−7^ and *P*_adj_ = 6.6*10^−6^ for pBC; *P*_adj_ = 9.4*10^−3^ and *P*_adj_ = 6.0*10^−9^ for mBC) were consistently significantly different after multiple testing correction (Fig. 3B, C; Additional file 1: Table S7). Intriguingly and similar to pBCs, none of the 18 CHEK2* mBCs displayed a somatic driver mutation in *TP53* (Fig. 3C). This mutual exclusivity between bi-allelic inactivation of *CHEK2* and somatic *TP53* mutations could suggest signaling of *CHEK2* c.1100delC through the TP53 pathway.

### Transcriptomics

**Fig. 4:**
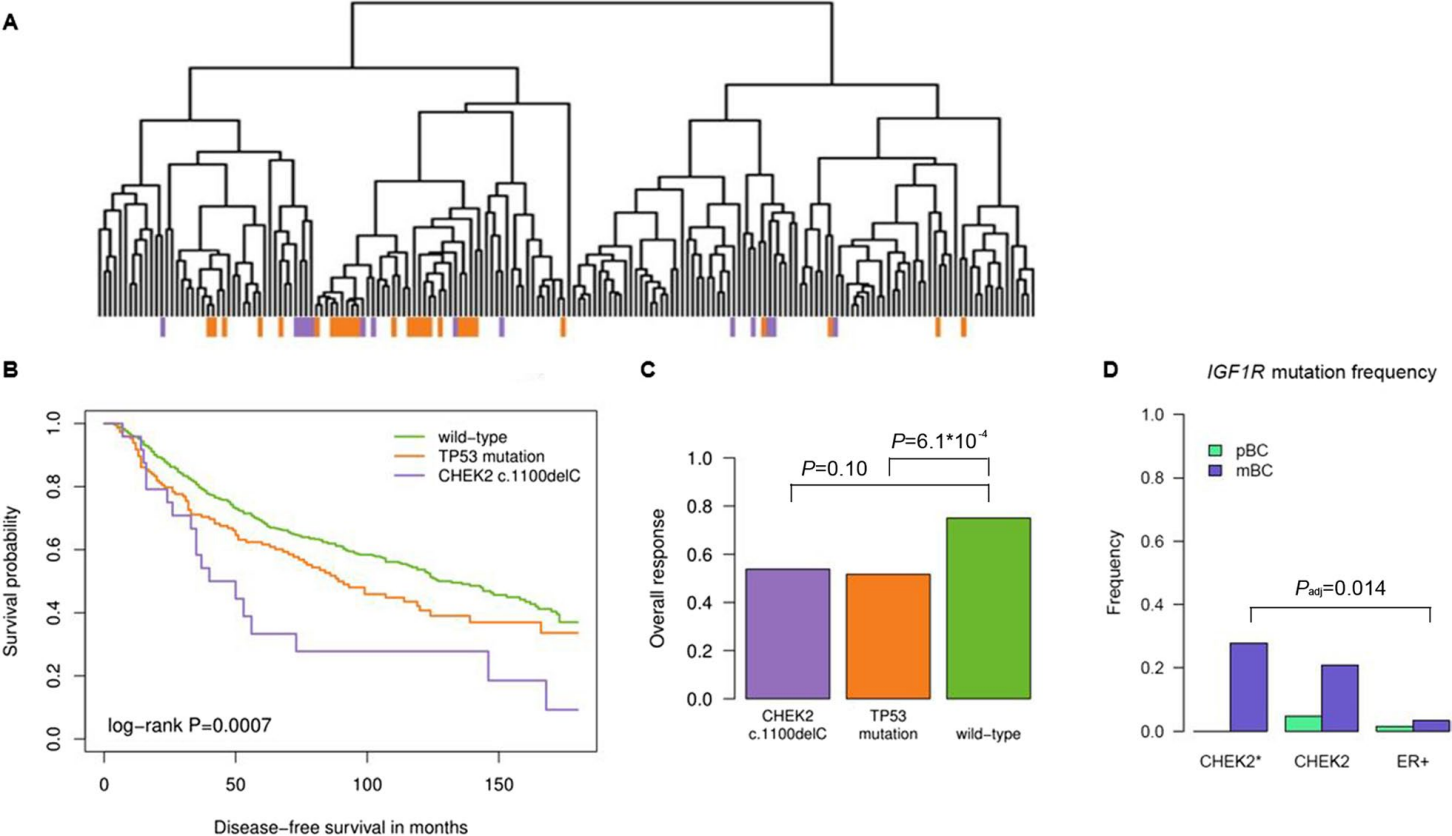
Transcriptomics, survival, tamoxifen therapy response and endocrine resistance mutations. **A** Hierarchical clustering of ER+ primary BCs (pBCs) based on 2,867 genes differentially expressed (regression model p-value < 0.05) between *TP53* mutant and wild-type ER+ pBCs. CHEK2* pBCs in purple and *TP53* mutant ER+ pBCs in orange. **B** Disease-free survival and **C** overall response to first-line tamoxifen among *CHEK2* c.1100delC mutation carriers, BC patients with a somatic *TP53* mutation and BC patients wild-type for both alleles. **D** Mutation frequency of the endocrine resistance gene *IGF1R* among CHEK2 and ER+ metastatic BCs

If the absence of somatic *TP53* mutations from CHEK2* BC genomes is a consequence of *CHEK2* c.1100delC signaling through the TP53 pathway, this should be discernible from the CHEK2 pBC transcriptomes (Additional file 3: Table S8). Therefore, we performed supervised clustering using 2,867 genes differentially expressed between *TP53* mutant vs wild-type ER+ pBCs from the ICGC cohort (Fig. 4A; Additional file 1: Table S9). The majority (9/14) of CHEK2* pBCs clustered among the *TP53* mutant-enriched cluster (*P* = 8.0*10^−5^), while the five remaining CHEK2* pBCs in the other cluster were close to the *TP53* mutant pBCs present there. Moreover, we found a 23-gene overlap between our 2,867 differently expressed genes and 31 genes from a previously published and widely used *TP53* gene signature [43]. This suggests that CHEK2* pBCs indeed have shared biology with *TP53*-mutated pBCs.

Next, to identify transcriptomic features exclusive to CHEK2* BCs, we extracted genes differentially expressed between CHEK2* and wild-type *TP53* pBCs (Additional file 2: Figure S3A). Among these 14 genes, no pathways were enriched (DAVID) [44] or known connections were discernable (STRING database) [45]. Moreover, we did not identify any overlap with the previously published 40-gene and 862-gene signatures of Nagel et al*.* and Muranen et al. [7, 19]. Of the 14 genes, *ATXN7* and *CDK5RAP3* have roles in DNA repair and these were downregulated in CHEK2* pBCs (Additional file 2: Figure S3B).

Thus, pBCs with bi-allelic loss of *CHEK2* share a common biology with *TP53* mutant pBCs, but no specific pathways were associated with the CHEK2-specific transcriptional profile itself.

### Survival and endocrine therapy resistance

Since *CHEK2* c.1100delC mutation carriers as well as patients with somatic *TP53* mutations have been shown to have unfavorable survival [5, 8, 10, 11, 46–49], we evaluated this among our retrospective cohorts of 760 lymph-node negative systemic treatment-naïve ER+ BC patients (prognostic cohort) and 323 hormone-naïve ER+ BC patients treated with first-line tamoxifen for recurrent disease (predictive cohort; clinicopathological variables in Additional file 1: Tables S10-11). Consistent with literature, *CHEK2* c.1100delC as well as *TP53* mutant BC patients had shorter disease-free survival (DFS) compared with BC patients wild-type for both alleles (CHEK2: HR = 2.26, 95% CI = 1.40–3.65, *P* = 8.2*10^−4^; TP53: HR = 1.30, 95% CI = 1.01–1.67, *P* = 0.039; Fig. 4B). After adjustment for classical prognostic factors, *CHEK2* c.1100delC appeared as an independent prognostic marker for DFS (HR = 2.23, 95% CI = 1.07–4.61, *P* = 0.031; Additional file 1: Table S12). In predictive analysis, *CHEK2* c.1100delC was not associated with response to tamoxifen in contrast to *TP53* mutations (overall response of 53.8% and 51.7% vs. 75% in wild-type; CHEK2: OR = 0.38, 95% CI = 0.13–1.20, *P* = 0.10; TP53: OR = 0.36, 95% CI = 0.20–0.64, *P* = 6.1*10^−4^; Fig. 4C). After adjustment for classical predictive factors, somatic *TP53* mutations remained independently associated with a poor response to tamoxifen treatment (OR = 0.42, 95% CI = 0.23–0.79, *P* = 7.1*10^−3^; Additional file 1: Table S13). Moreover, in the prognostic and predictive cohort combined (*n* = 942; *n* = 141 BC patients in both cohorts), none of the 11 patients with bi-allelic inactivation of *CHEK2* had a *TP53* mutation (*P* = 0.14), again confirming what we observed among pBC and mBC genomes.

We also evaluated mutation frequencies of 23 genes associated with endocrine resistance in CHEK2 versus ER+ mBC genomes (Additional file 1: Table S14). Interestingly, the greatest increase in mutation frequency for CHEK2 mBC compared with pBC was observed for the *IGF1R* gene (0% vs. 27.8%, *P*_nom_ = 0.052, *P*_adj_ = 1). Moreover, out of these 23 genes, *IGF1R* was the only gene for which the mutation frequency was significantly different between CHEK2 and ER+ mBCs (27.8% vs. 3.3%, *P*_adj_ = 0.014) and *IGF1R* mutations (mostly amplifications) associated with an elevated gene expression in a subset of 127 mBCs for which we had RNAseq data (*P* = 0.080). However, when we combined all genes, no difference in the frequency of CHEK2 vs. ER+ mBCs with either one or multiple resistance mutations was observed (94.4% vs. 91.1%, *P* = 1).

Thus, we confirmed in a third independent cohort that BCs with bi-allelic loss of *CHEK2* do not harbor somatic *TP53* mutations and that the unfavorable survival of *CHEK2* c.1100delC carriers is likely driven by intrinsic tumor aggressiveness rather than endocrine resistance.

### WGD and chromothripsis

**Fig. 5:**
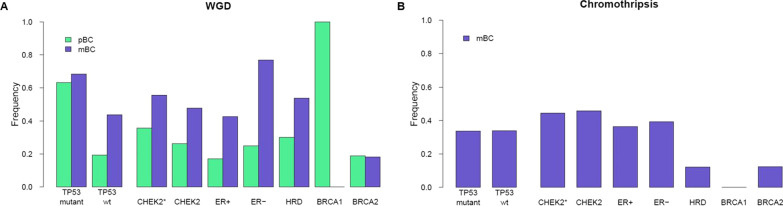
WGD and chromothripsis. **A** Frequency of WGD in primary and metastatic BC. For the subgroup frequencies, only *TP53* wild-type cases were included. **B** Chromothripsis frequencies among subgroups of metastatic BC patients

Since WGD is 1.8-fold more common in BC genomes with somatic *TP53* mutations [50], we also evaluated WGD among CHEK2 BC genomes. In pBCs, 143/226 (63.3%) *TP53* mutant BCs had WGD compared with 62/321 (19.3%, *P* = 2.2*10^−16^) *TP53* wild-type BCs (Fig. 5A). Interestingly, the WGD frequency of CHEK2 pBCs was in between *TP53* wild-type and mutant pBCs (35.7% vs. 19.3% and 63.3%, respectively, *P* = 0.17 and *P* = 0.049), which fits the moderate BC risk associated with *CHEK2* c.1100delC. Other subgroups, including only *TP53* wild-type pBCs, had lower WGD frequencies than CHEK2* pBCs (*i.e.,* 18.2% combined, *P* = 0.15), except for BRCA1 pBCs (all four showed WGD, Fig. 5A), although this was not significant. Interestingly, WGD frequency increased 1.5 to twofold in *TP53* wild-type, CHEK2*, HRD, ER+ and ER− mBCs as compared with pBCs, but not for *TP53* mutant and BRCA2 mBCs (disregarding the single BRCA1 mBC). Regardless, WGD frequency of CHEK2* mBCs was again in between *TP53* wild-type and mutant mBCs (55.6% vs. 43.8% and 68.5%, respectively, *P* = 0.46 and *P* = 0.30; Fig. 5A).

Chromothripsis, a single catastrophic event of clustered SVs, has also been associated with *TP53* mutation [51]. Unfortunately, due to low resolution, identifying chromothripsis using the publicly available CN and SV data of ICGC was not possible. In CHEK2 pBCs, however, the chromothripsis frequency was 33.3%, which increased to 44.4% in the mBCs (Fig. 5B). Also, CHEK2 mBCs more frequently displayed chromothripsis than HRD+ mBCs (44.4% vs. 11.7%, *P* = 4.5*10^−3^), but not compared to ER+ and ER− mBCs (44.4% vs. 36.5% and 39.2%, *P* = 0.62 and *P* = 0.79; Fig. 5B). Intriguingly, although chromothripsis was most frequent among CHEK2* mBCs, we could not replicate the association between *TP53* mutations and chromothripsis in mBCs (*P* = 1), however chromothripsis was associated with WGD (*P* = 4.6*10^−3^).

Thus, both WGD and chromothripsis increased with disease progression for CHEK2* BCs. Moreover, CHEK2* BCs had a WGD frequency intermediate to wild-type and *TP53* mutant BCs and the highest frequency of chromothripsis compared with other mBC groups.

### Structural variant size distribution

**Fig. 6:**
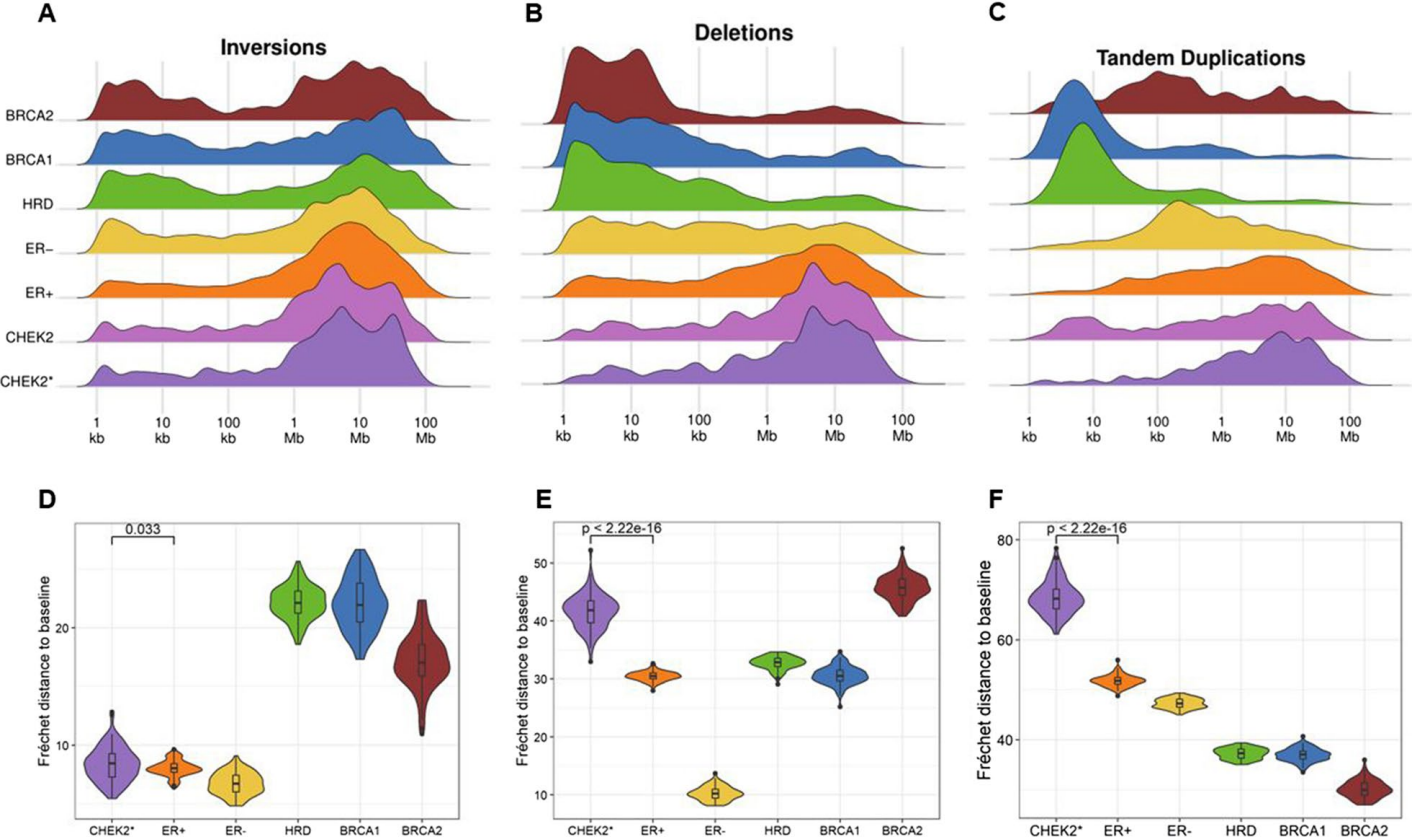
SV size distributions among primary BC subgroups. **A-C** SV size density profiles of and **D-F** Fréchet distances to baseline for inversions, deletions and tandem duplications (left to right)

We also interrogated SV sizes among CHEK2 pBCs and mBCs (Additional file 1: Table S15). For inversions, both ER+ and CHEK2 pBCs displayed less small (< 100 kb) inversions than other groups. Larger (> 100 kb) inversions, however, were seen in all groups although size distribution patterns varied among groups. Interestingly, CHEK2 pBCs displayed two specific peaks (at 5.6 and 28.2 Mb), whereas large SV sizes in ER+ pBCs were normally distributed (Fig. 6A). We evaluated differences between SV profiles more precisely by calculating the Fréchet distance (FD) of each group’s inversion profile to the inversion profile of all samples combined (*i.e.,* the baseline). The size distribution (after 100 bootstraps) of inversions in CHEK2* pBCs was most comparable to ER+ pBCs, but still significantly different (mean FD from baseline of 8.38 vs. 8.04, *P* = 0.033 Fig. 6D). This observation was validated in mBCs (mean FD from baseline of 6.61 vs. 4.72, *P* < 2.2*10^−16^; Additional file 2: Figure S4A).

Regarding deletions, HRD+ pBCs predominantly displayed deletions < 500 kb in size, whereas ER+ and CHEK2 pBCs mostly displayed deletions > 500 kb. Moreover, CHEK2 pBCs specifically displayed two peaks at 4.5 and 28.2 Mb, whereas ER+ pBCs displayed one broad peak with the most frequent deletion size around 8.9 Mb (Fig. 6B). Similar to inversions, the deletion size distribution was significantly different between CHEK2 and ER+ groups, both in pBCs and mBCs (pBC: mean FD from baseline of 41.66 vs. 30.47, *P* < 2.2*10^−16^ (Fig. 6E); mBC: 16.81 vs. 18.59, *P* = 3.5*10^−14^ (Additional file 2: Figure S4B).pBCs also displayed varying size distribution profiles for tandem duplications (TDs) among groups. Specifically, BRCA1 and HRD pBCs predominantly displayed smaller (< 100 kb) TDs, whereas ER− and BRCA2 pBCs mostly displayed intermediate size (50–500 kb) TDs. Larger (> 500 kb) TDs were predominantly observed for ER+ and CHEK2 pBCs. Interestingly, ER+ pBCs again displayed one broad peak, whereas CHEK2 pBCs displayed multiple peaks most prominently at 8.9 and 22.4 Mb (Fig. 6C). Consequently, also the TD size distribution was significantly different between CHEK2 and ER+ pBCs (mean FD from baseline of 68.47 vs. 51.80, *P* < 2.2*10^−16^; Fig. 6F) and mBCs (mean FD from baseline of 33.15 vs. 23.97, *P* < 2.2*10^−16^; Additional file 2: Figure S4C).

Taken together, CHEK2 pBCs display a unique size distribution profile of inversions, deletions and TDs, unlike any of the other pBC subgroups. Importantly, this SV size distribution profile could not be replicated by randomly subsampling SVs from ER+ pBC genomes (Additional file 2: Figure S5) indicating these findings are not a result of the smaller sample size of CHEK2 BCs. Moreover, the relatively high chromothripsis frequency in CHEK2* mBCs did not appear to be causal for the CHEK2 size distribution profile. Although TDs located in the CHEK2-specific peaks were more frequently located inside chromothriptic regions (*P* = 8.3*10^−3^), this was not the case for inversions and deletions (*P* = 0.71 and *P* = 0.12, respectively), nor for TDs located in the ER+ specific peaks (*P* = 0.87).

## Discussion

Our interrogation of the somatic landscape of CHEK2 BCs revealed novel genomic features specific to CHEK2-driven BC. First, and in agreement with Mandelker et al*.*, we did not observe an HRD phenotype among CHEK2 BCs [26]. Instead, CHEK2 BCs were most similar to ER+ BCs. Second, CHEK2 BC genomes that lost the wild-type *CHEK2* allele did not harbor any somatic *TP53* mutations (*i.e.,* 0/43 in all three cohorts combined). Third, CHEK2* BCs displayed a unique size distribution of SVs that is not simply caused by the increased chromothripsis frequency among these genomes.

There are two reasons why the latter two observations were not reported by Mandelker et al*.*, which also represent strengths of our study. First of all, inherent to the nature of their data (from whole exome sequencing and targeted sequencing using the MSK-IMPACT panel) structural variation and related events such as chromothripsis could not be evaluated. Second, although Mandelker et al*.* evaluated allelic loss at the *CHEK2* locus, they instead opted to stratify samples according to low and high-risk *CHEK2* variants. Since our cohort consisted only of BCs from c.1100delC carriers, we did not have to prioritize classification in this respect. Another strength of our study was the availability of a second cohort for validation purposes. A disadvantage of having an mBC cohort for validation, however, is that due to disease progression and/or treatment-induced selection meaningful pBC-specific associations could have been obfuscated.

Our observation that CHEK2* pBCs do not harbor any somatic *TP53* mutations and have at least part of their biology in common with *TP53* mutant pBCs may not be completely surprising. Several studies in the past have found links between inactivation of CHEK2 and TP53 pathway signaling during tumorigenesis. However, results have often been conflicting, thus placing doubts on their validity. For example, in thymocytes from two different *Chk2*^−/−^ mouse models Chk2 seemed to regulate p53-dependent apoptosis [14–16], but this was not confirmed in a knock-in *Chk2* c.1100delC mouse model [17]. Moreover, before *CHEK2* c.1100delC was identified to be a moderate-risk BC susceptibility gene, it was actually a candidate gene for Li-Fraumeni syndrome [1, 2, 52, 53], which is caused by germline mutations in *TP53* [54, 55]. More recently, Boonen et al*.* identified CHEK2-dependent phosphorylation of KAP1 p.S473 to be an excellent functional read-out for pathogenicity of germline *CHEK2* variants [56]. Interestingly, KAP1 is a nuclear co-repressor that inactivates TP53 [57]. Unfortunately, despite many links observed between CHEK2 and TP53, how precisely *CHEK2* c.1100delC could promote tumorigenesis through the TP53 pathway is still unclear. For this, functional studies in proper model systems (*i.e.,* ER+ human breast cells) are required.

Further supporting the shared biology between CHEK2* and *TP53* mutant BCs is the observation that CHEK2* pBCs had the highest WGD frequency among the subgroups, a feature enriched among *TP53* mutant cancers [50]. In fact, WGD frequency of CHEK2* genomes was intermediate to *TP53* wild-type and mutant BCs, an observation fitting the incomplete penetrance of *CHEK2* c.1100delC. Considering the many roles of TP53 as well as CHEK2, and only a subset overlapping, not all roles these proteins fulfil will be relevant for tumorigenesis. Consistent though, with the high WGD frequency among CHEK2* pBCs, embryonic fibroblasts from knock-in *Chk2* c.1100delC mice showed an altered cell cycle distribution and a population of cells that are multinuclear, indicative of a cytokinesis defect [17]. It may thus be interesting to subclassify WGD-positive cancers in those being multinucleated versus polyploid, since underlying causal mechanisms and thus players involved may be different.

Lack of somatic *TP53* mutation among CHEK2* BC genomes may also be interpreted as a lack of severity of *CHEK2* c.1100delC-driven BC instead of signaling through the TP53 pathway. However, consistent with literature [5, 8, 10, 11, 46–49], we observed that BC patients with germline *CHEK2* c.1100delC or a somatic *TP53* mutation have an unfavorable clinical outcome compared to wild-type patients. In fact, we here show that *CHEK2* c.1100delC is an independent prognostic factor, whereas *TP53* mutation is an independent predictor of response to tamoxifen. This is in agreement with two previous studies showing the efficacy of chemotherapy or endocrine therapy is unlikely to account for the unfavorable survival of *CHEK2* mutation carriers [11, 12]. However, considering the small group of *CHEK2* mutation carriers in the predictive cohort (*n* = 13) and the similar overall response rates in *CHEK2* mutation carriers and patients with *TP53* mutations, power could have been an issue in this analysis. If proven irreproducible, *IGF1R* could be an endocrine resistance gene to investigate further since *IGF1R* overexpression has been associated with poor outcome and resistance to conventional BC therapies [58].

Another key finding from our analyses was that CHEK2 BCs display a unique size distribution of SVs, most similar to, but significantly different from ER+ BCs. Considering previous reports associating genes with a specific SV size distribution, size distribution profiles can also be considered biological scars arising from specific mutational events. For example, combined inactivation of *TP53* and *BRCA1* produced TDs with an average length of 11 kb, while CCNE1 pathway activation and *CDK12* mutations generated TDs with an average length of 231 kb and 1.7 Mb, respectively [59]. In addition, deletions in metastatic colorectal cancers were predominantly 10 kb to 1 Mb in size and frequently located in common fragile sites. Further analyses of breakpoints and localization of these deletions suggested transcription-dependent double-fork failure as an origin [60]. Therefore, unravelling the underlying mechanism that generates the CHEK2-specific SV size distribution profile would be an important aspect of understanding how *CHEK2* c.1100delC promotes breast tumorigenesis. Despite the high chromothripsis frequency among CHEK2* BCs, chromothripsis did not appear to be the (sole) driver of the CHEK2-specififc SV size distribution profile. Also, a mechanistic overlap with previously published size distribution patterns is not evident [59, 60].

CHEK2* BCs were most similar to ER+ BCs, even indistinguishable in some aspects, suggesting overlapping tumor evolution. Still, *CHEK2* c.1100delC carriers have a shorter survival and intrinsic tumor aggressiveness plays a role. To provide efficacious anti-cancer treatment and chemoprevention for these women, we need to identify the Achilles’ heel for CHEK2-driven tumorigenesis. We and others have by now firmly established that CHEK2 BCs do not display HRD and thus *CHEK2* mutation carriers will not benefit from PARP inhibitor therapy [26, 61–63]. Moreover, because of the relatively low TMB we observed among CHEK2 BCs, these women are also not likely to benefit from immune checkpoint inhibitor therapy, but clinical trials investigating this are needed. The CHEK2-specific genomic features we identified here should therefore be further interrogated *in silico* as well as propel further functional experiments to finally unravel the mechanism of CHEK2-driven tumorigenesis, thereby paving the way for personalized medicine for *CHEK2* mutation carriers.

## Conclusions

CHEK2 BC genomes were most similar to non-HRD, ER+ BC genomes in terms of TMB, ID ratio as well as the various mutational signatures, yet they display a worse prognosis likely originating from an increased intrinsic tumor aggressiveness. Unfortunately, considering HRD status as well as TMB, *CHEK2* mutation carriers are not likely to benefit from either PARP inhibitors or immune checkpoint inhibitors. Importantly, CHEK2 BC* genomes did not harbor somatic *TP53* mutations and displayed similar biology as *TP53* mutant BCs. Moreover, CHEK2* BC genomes display a unique size distribution of SVs that is not simply caused by the increased chromothripsis frequency among these genomes. These findings provide novel clues for unraveling the mechanisms of CHEK2-driven tumorigenesis.

## Supplementary Information

**Additional file 1: Table S1.** Characteristics of breast cancers from CHEK2 c.1100delC mutation carriers. **Table S2.** Number of variants, TMB, ID ratio, TP53 status, WGD, chromothripsis and percentage relative contribution of SBS, DSB, ID and SV signatures for primary and metastatic breast cancer genomes. **Table S3.** Relative contribution of major single base substitution signatures in primary and metastatic breast cancer genomes. **Table S4.** Relative contribution of major doublet base substitution signatures in primary and metastatic breast cancer genomes. **Table S5.** Relative contribution of major small indel signatures in primary and metastatic breast cancer genomes. **Table S6.** Relative contribution of 6 known structural variant signatures in CHEK2 versus HRD, ER− and ER+ primary breast cancer genomes. **Table S7** Somatic driver gene mutation frequencies in primary and metastatic breast cancer genomes. **Table S9.** Genes differentially expressed between TP53 mutant and wild-type pBCs. **Table S10.** Clinicopathological variables of 760 ER+ lymph node negative treatment-naive breast cancer patients. **Table S11.** Clinicopathological variables of 323 hormone-naïve ER+ breast cancer patients treated with first-line tamoxifen for recurrent disease. **Table S12.** Univariable and multivariable Cox regression analysis of disease-free survival in 760 ER+ lymph node negative treatment-naive breast cancer patients. **Table S13.** Univariable and multivariable logistic regression analysis of overall response in 323 hormone-naïve ER+ breast cancer patients treated with first-line tamoxifen for recurrent disease. **Table S14** Endocrine therapy resistance gene mutation frequencies in metastatic breast cancer genomes. **Table S15.** Sizes of structural variant types in primary and metastatic breast cancer genomes.

**Additional file 2: Figure S1.** Mutational signatures among metastatic BC genomes. **Figure S2.** Relative contribution of mutational signatures in CHEK2* and ER+ pBC genomes. **Figure S3.** Genes differentially expressed between CHEK2* versus TP53 wild-type ER+ pBCs. **Figure S4.** Distribution of Fréchet distances among the subgroups of mBC genomes. **Figure S5.** Subsampling SVs from ER+ pBC genomes.

**Additional file 3: Table S8.** RNA sequencing log2 GeTMM values from CHEK2 pBCs.

## Data Availability

Somatic genomic features and RNA sequencing data from CHEK2 pBCs generated for this study are included in this published article and its supplementary information files. The previously published pBC genome dataset generated by the ICGC [23] is available in the European Genome-phenome Archive under accession code EGAS00001001178. The pre-existing mBC genome dataset was generated by the CPCT [29] and obtained from HMF under data request DR-085.

## Abbreviations

APOBEC: Apolipoprotein B mRNA editing enzyme, catalytic polypeptide
ATM: Ataxia telangiectasia-mutated
BC: Breast cancer
BRCA1: Breast cancer type 1 susceptibility protein
BRCA2: Breast cancer type 2 susceptibility protein
CCND1: Cyclin D1
CCNE1: Cyclin E1
CDC25A: Cell division cycle 25 homolog A
CDK12: Cyclin-dependent kinase 12
CHEK2: Checkpoint kinase 2
CN: Copy number
CPCT: Center for Personalized Cancer Treatment
DBS: Double base substitution
DFS: Disease-free survival
DDR: DNA damage response
ER: Estrogen receptor
FD: Fréchet distance
HMF: Hartwig Medical Foundation
HR: Hazard ratio
HRD: Homologous recombination repair deficient
HRP: Homologous recombination repair proficient
ICGC: International Cancer Genome Consortium
IGF1R: Insulin growth factor 1 receptor
ID: Insertion/deletion
Kb: Kilo base
Mb: Mega base
mBC: Metastatic breast cancer
MNV: Multi nucleotide variant
OR: Odds ratio
PALB2: Partner and localizer of BRCA2
PARP: Poly ADP ribose polymerase
pBC: Primary breast cancer
PML: Promyelocytic leukemia protein
SBS: Single base substitution
SNV: Single nucleotide variant
SV: Structural variant
TD: Tandem duplication
TMB: Tumor mutational burden
TP53: Tumor protein p53
WGD: Whole-genome duplication
WGS: Whole-genome sequencing
