# Supplementary material for: Breast cancer genomes from CHEK2 c.1100delC mutation carriers lack somatic TP53 mutations and display a unique structural variant size distribution profile
Source: Breast Cancer Res. 2023 May 9;25:53. doi: 10.1186/s13058-023-01653-0 (PMC10169359; doi:10.1186/s13058-023-01653-0)
Supplement: Supplementary file 2 — Additional file 2: Figure S1. Mutational signatures among metastatic BC genomes. Figure S2. Relative contribution of mutational signatures in CHEK2* and ER+ pBC genomes. Figure S3. Genes differentially expressed between CHEK2* versus TP53 wild-type ER+ pBCs. Figure S4. Distribution of Fréchet distances among the subgroups of mBC genomes. Figure S5. Subsampling SVs from ER+ pBC genomes. [file 13058_2023_1653_MOESM2_ESM.pdf]

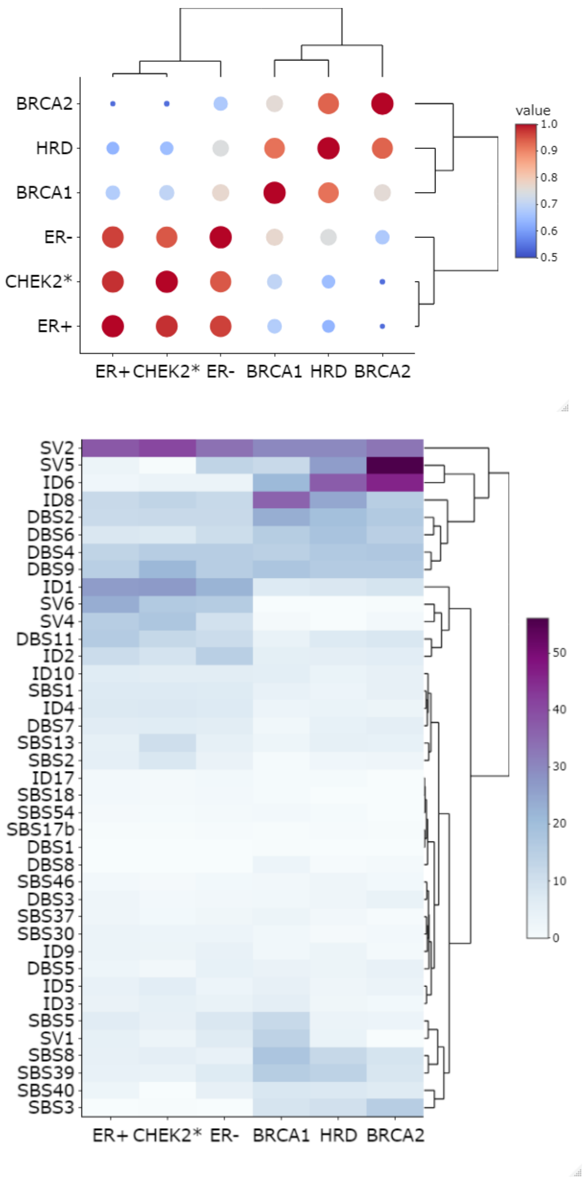

Figure S1. Mutational signatures among metastatic BC genomes. Cosine similarity coefficients (top) and hierarchical clustering (bottom) of breast cancer subgroups based on the median relative contribution of 39 SBS, DBS, ID and SV signatures.

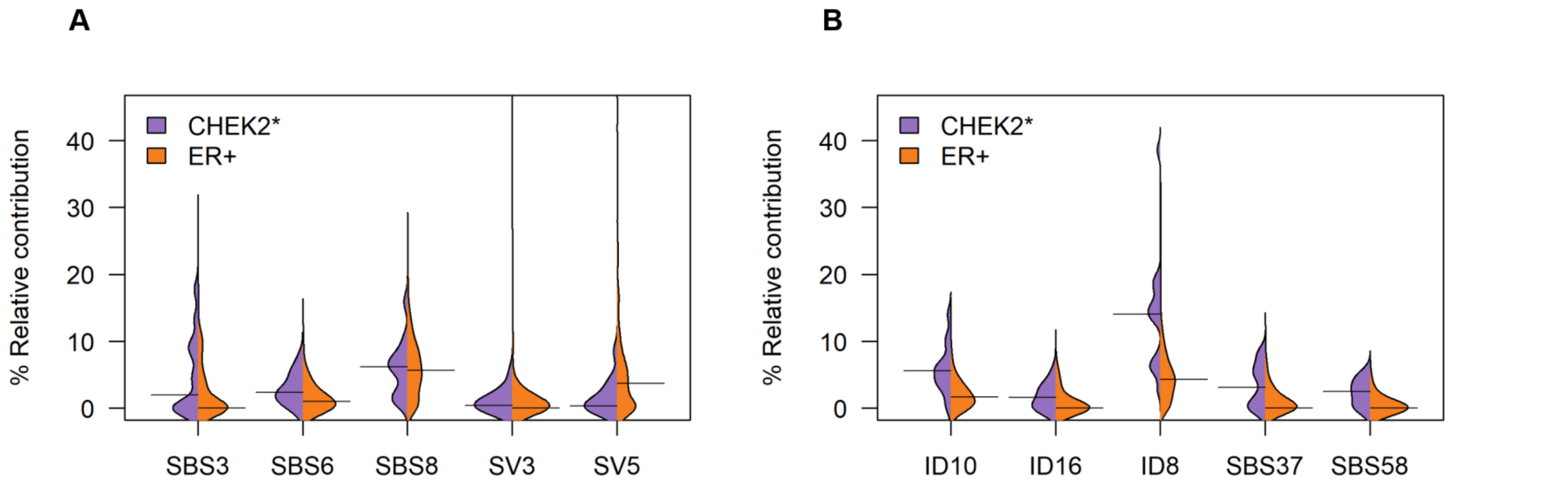

Figure S2. Relative contribution of mutational signatures in CHEK2* and ER+ pBC genomes**. A** HRD-specific signatures. **B** Signatures significantly different between CHEK2* and ER+ pBCs. Horizontal lines indicate median levels.

A

ENSG-ID	name	Coefficient	P-value
ENSG00000163635	ATXN7	0.3472	0.002
ENSG00000149531	FRG1B	0.7715	0.021
ENSG00000086848	ALG9	0.3416	0.021
ENSG00000163633	C4orf36	0.3889	0.026
ENSG00000236824	BCYRN1	0.6915	0.028
ENSG00000104728	ARHGEF10	0.4494	0.033
ENSG00000110536	PTPMT1	-0.3408	0.035
ENSG00000108465	CDK5RAP3	0.4819	0.040
ENSG00000243646	IL10RB	0.3312	0.041
ENSG00000204822	MRPL53	-0.3091	0.041
ENSG00000144619	CNTN4	1.0656	0.043
ENSG00000175691	ZNF77	-0.2505	0.044
ENSG00000160408	ST6GALNAC6	0.4287	0.046
ENSG00000221930	FAM45B	0.3725	0.049

B

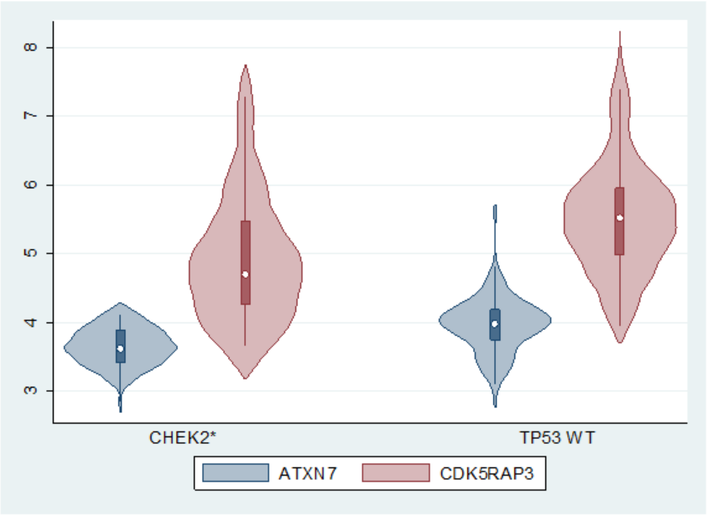

Figure S3. Genes differentially expressed between CHEK2* versus *TP53* wild-type ER+ pBCs. **A** 14 genes that were differentially expressed. **B** Expression levels (log2) of *ATXN7* and *CDK5RAP3* among the two groups. White circles indicate median levels.

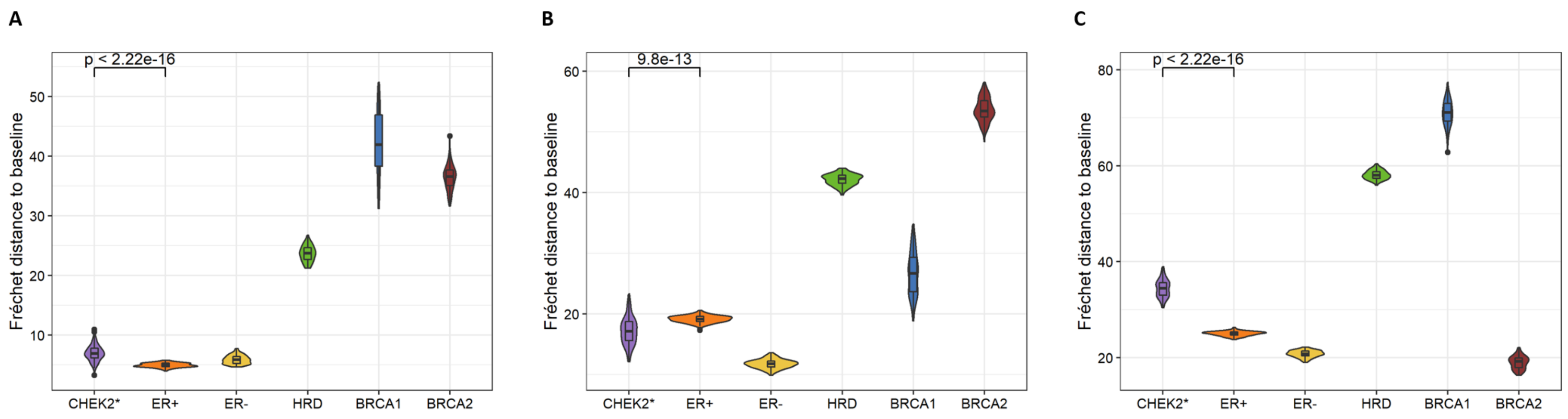

Figure S4. Distribution of Fréchet distances among the subgroups of mBC genomes for **A** inversions, **B** deletions and **C** tandem duplications.

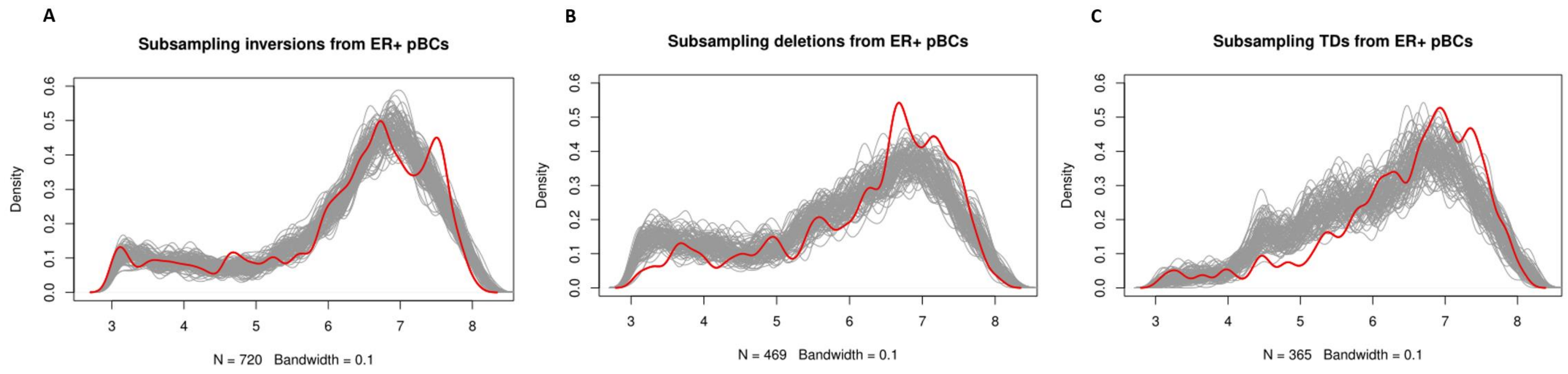

Figure S5. Subsampling SVs from ER+ pBC genomes. Grey density curves represent 100 subsampling iterations from ER+ pBCs, whereas the red density curve represents the CHEK2* pBCs. **A** Inversions, **B** deletions, **C** tandem duplications.
